# Intravascular large B-cell lymphoma of the central nervous system with renal involvement: a case report and literature review

**DOI:** 10.3389/fonc.2025.1502574

**Published:** 2025-01-30

**Authors:** Jun Li, Zhaojiao Li, Yifeng Shi, Jiajie Chen, Heng Zhao, Xueye Mao, Shan Li, Huiying Wang, Qiang Meng, Lingchun Liu

**Affiliations:** ^1^ Department of Neuology, The Affiliated Hospital of Kunming University of Science and Technology, The First People’s Hospital of Yunnan Province, Kunming, China; ^2^ Department of Neurosurgery, The Affiliated Hospital of Kunming University of Science and Technology, The First People’s Hospital of Yunnan Province, Kunming, China

**Keywords:** intravascular large B-cell lymphoma, central nervous system vasculitis, MRI imaging features, pathology biopsy, differential diagnosis

## Abstract

Intravascular large B-cell lymphoma (IVLBCL) is a highly aggressive type of non-Hodgkin’s lymphoma. The clinical manifestations and imaging of this disease are nonspecific, making diagnosis challenging. We report a case of a patient with recurrent stroke-like symptoms, primarily characterized by hemiplegia, vertigo, ataxia, and proteinuria. Brain MRI revealed multiple cerebral infarctions, microbleeds, and meningeal enhancement. Ultimately, the patient was diagnosed with IVLBCL through a brain tissue biopsy, and involvement of the kidneys was suspected. We suggest considering IVLBCL in patients who present with recurrent stroke-like symptoms, fluctuating neurological deficits, the aforementioned MRI findings, and involvement of other organs. Additionally, central nervous system vasculitis(CNSV) may represent a transitional manifestation of the disease. Pathological biopsy is the gold standard for diagnosis. We hope that through this case, doctors can gain a deeper understanding of IVLBCL, enabling early diagnosis and timely treatment to improve prognosis.

## 1 Introduction

IVLBCL is a common type of intravascular lymphoma. As a rare form of extranodal non-Hodgkin’s lymphoma, it is characterized by the selective proliferation and abnormal aggregation of tumor-like lymphocytes within small and medium blood vessels, leading to high invasiveness. The incidence of IVLBCL is less than 1 in 1,000,000, with a mortality rate exceeding 80% and a three-year overall survival rate of only 17%. The disease lacks specific manifestations, making it difficult to diagnose and differentiate from other conditions. Currently, the gold standard for diagnosis is pathological tissue biopsy. In this report, we explore the laboratory examinations, imaging findings, and the relationship with CNSV, along with diagnostic methods for the disease. Our goal is to facilitate early identification of the disease in clinical practice.

## 2 Case presentation

A 51-year-old woman was admitted to the hospital on November 16, 2023, due to persistent dizziness, nausea, and vomiting for over six months. She had three previous episodes in April, August, and September. The patient’s symptoms initially appeared acutely in April 2023. There was no identifiable trigger for her dizziness, which was accompanied by headache, vomiting, walking instability, numbness in the right upper limb, and a skewed mouth. A head Magnetic resonance imaging(MRI) performed at a local hospital revealed multiple lesions in the frontal, temporal, and parietal regions. The cerebrospinal fluid routine and biochemistry from a lumbar puncture were normal, and there were no abnormalities in the head and neck computed tomography angiography(CTA). Considering the diagnosis of cerebral infarction, the patient was prescribed aspirin (100 mg), clopidogrel (75 mg), and atorvastatin (20 mg). The symptoms and signs of the subsequent two episodes were similar, and the patient received the same treatment as before. The most recent occurrence in November involved aggravated dizziness and headache, accompanied by visual rotation, nausea, vomiting, and an inability to stand or walk. Throughout the course of her illness, the patient did not experience fever, seizures, disturbances in consciousness, or mental and behavioral abnormalities, but she did lose 8 kg in weight.

The physical examination showed no signs of facial paralysis. The pupils were equal and round, light reflexes were present, limb muscle strength was rated at grade 5, tendon reflexes were symmetrical, and no bilateral pathological signs were observed. The patient exhibited symmetrical sensation. The heel-knee-tibia test showed bilateral instability. The patient had difficulty standing with eyes closed and could not cooperate during the examination. Neck resistance was noted with two horizontal fingers and the national institutes of health stroke scale (NIHSS) score was 2.

**Figure 1:**
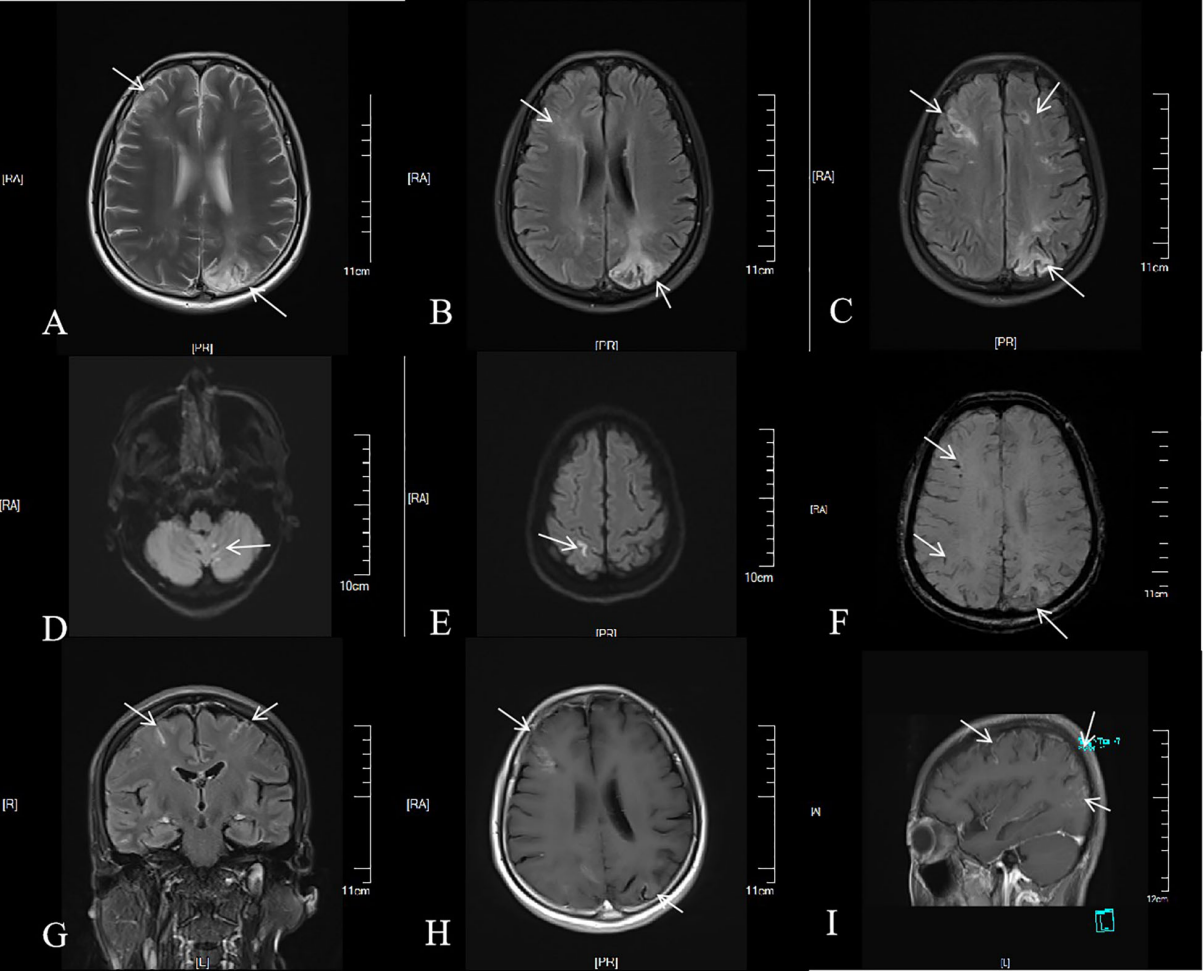
Brain MRI of the patient. Multiple patchy long T2 signals were seen in the cortex, subcortex and white matter of the right frontal lobe of the left occipital lobe **(A)**. On FLAIR sequence, multiple punctate and patchy high signals were seen in the cortex, subcortex and white matter of the bilateral frontal, parietal and occipital lobes **(B, C)**. Cerebellar vermis punctate, left parietal lobe linear high signal on DWI; b = 1,000 **(D, E)**. Multiple low signals were found in the left frontal lobe, parietal lobe and right occipital lobe on SWI **(F)**. Linear enhancement signals were seen in the pia mater, left occipital lobe, right frontal lobe, bilateral parietal lobe, cortex and subcortex **(G, H)**, and circular enhancement signals were seen in the sagittal subcortex and white matter **(I)**.

**Figure 2:**
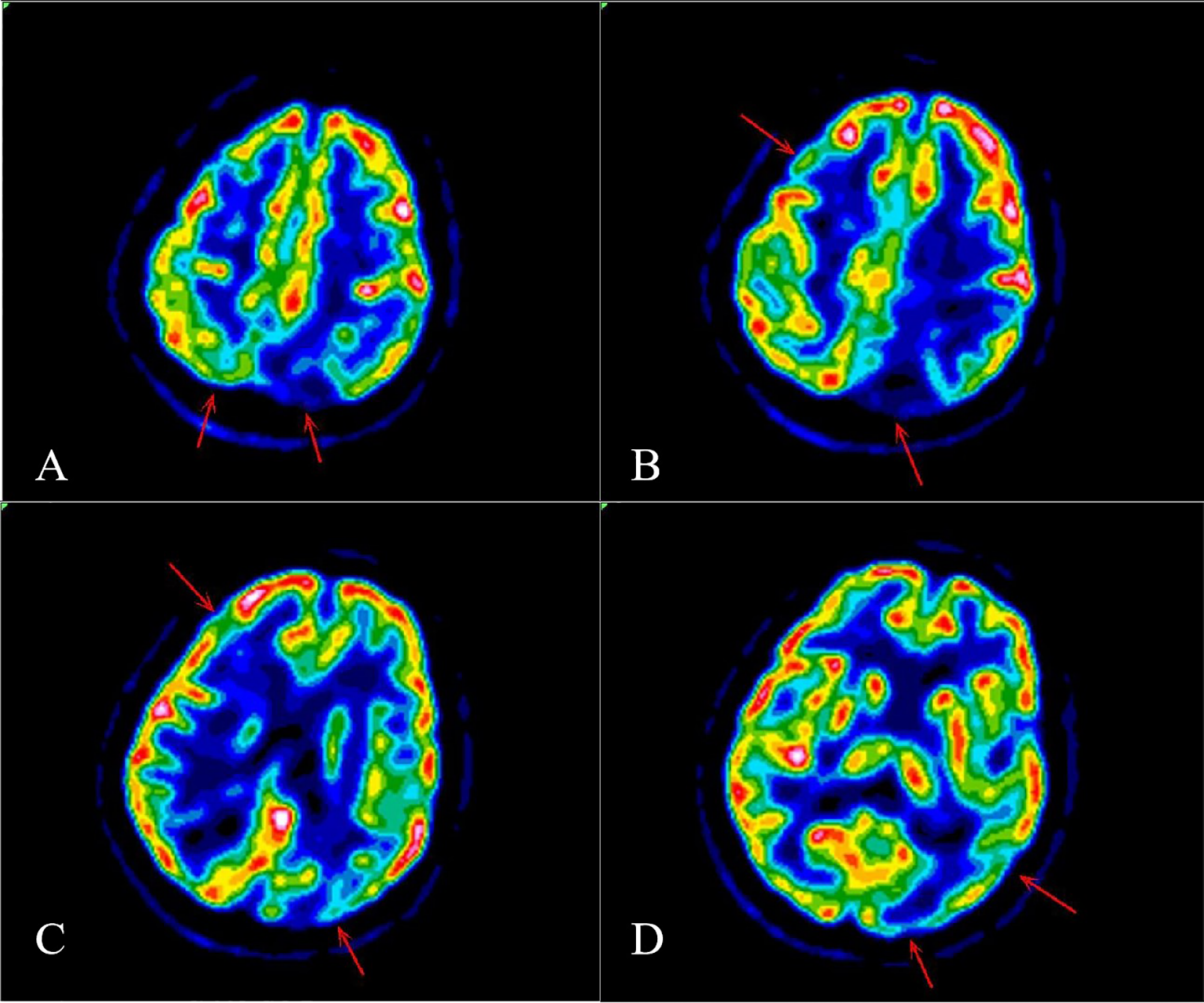
PET-CT showed decreased cortical metabolism in the right frontal lobe, parietal lobe, occipital lobe and left temporal lobe **(A-D)**.

**Figure 3:**
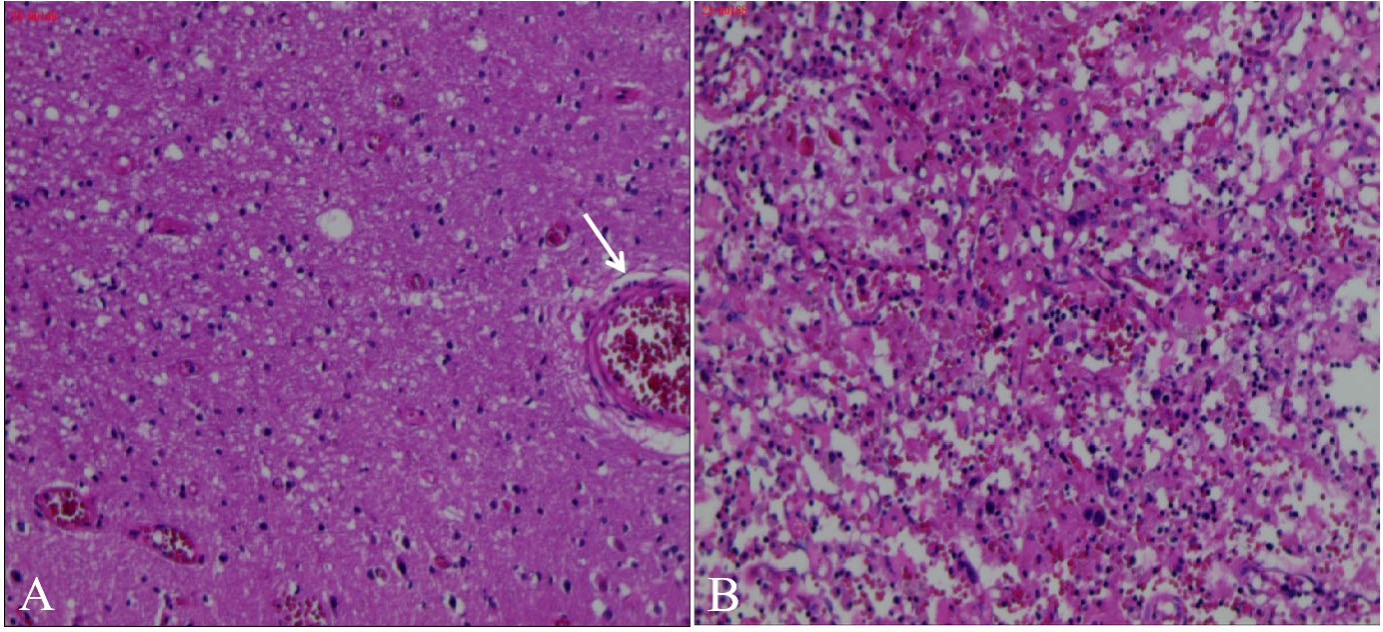
Pathological biopsy of occipital lobe of brain. H&E stain in our patient showed Multiple deep stained cell aggregation in blood vessels and partly outside the blood vessels on light microscope **(A)**, adjusted to 800x, many round and darkly stained cells were found to aggregate **(B)**.

**Figure 4:**
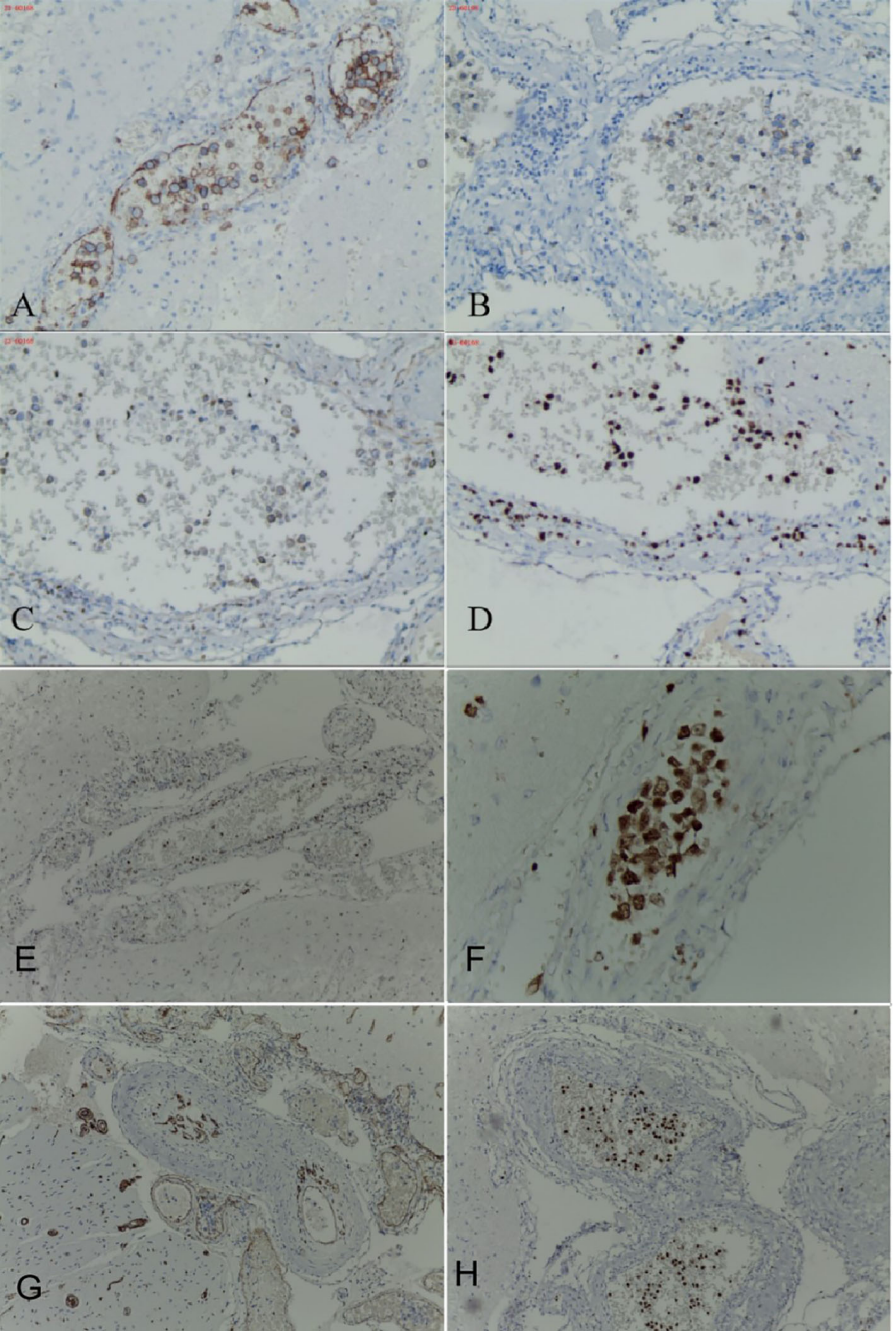
Occipital lobe immunohistochemistry of the patient, stained tumor cells gathered in the blood vessels. Occipital cortical artery positive for CD20 **(A)**, occipital cortical artery positive for CD19 **(B)**, occipital cortical artery partially positive for Bcl-2 **(C)**, occipital cortical artery positive for Ki-67,proliferation index 60% **(D)**, occipital cortical artery positive for Bcl-6 **(E)**, occipital cortical artery positive for Mum-1 **(F)**, occipital cortical artery positive for CD34 **(G)**, occipital cortical artery positive for Pax-5 **(H)**.

The blood antinuclear antibody(ANA) was 1:80 (normal is <1:40), blood urea nitrogen(BUN) was range of 6.79-8.6 mmol/L(normal range:2.6-7.5mmol/L),the first BUN was 8.6 mmol/L, creatinine(Cr)was range of 70-107 umol/L(normal range:41-73umol/L),the first Cr was 77umol/L,blood albumin(ALB) was range of 32.7-42.0 g/L(normal range:40-55g/L),the first ALB was 39.4 g/L and the urine analysis showed a protein level of 3+ with a total protein excretion of 669 mg over 24 hours(normal range:0-300mg). The cerebrospinal fluid(CSF) examination revealed a colorless and clear appearance. The CSF pressure was measured at 100 mmH_2_O, 2 ml of CSF was within cell count of 7 × 10^6^/L(normal range: 0-10 ×10^6^/L), consisting of 65% lymphocytes, 30% monocytes, 3% activated lymphocytes, and 2% other activated lymphocytes. The CSF protein concentration was 476 mg/L (normal range: 150-450 mg/L), and glucose level was 5.2 mmol/L (normal range: 2.2-3.9 mmol/L). The CSF immunoglobulin levels were IgG at 60.0 mg/L (normal range: 0-34 mg/L) and IgA at 9.3 mg/L (normal range: 0-5 mg/L). The CSF ANA was 1:100(normal is negative).Other laboratory test results including Procalcitonin(PCT), C-reactive protein(CRP), antiphospholipid antibody(APLA), tumor markers, and disseminated intravascular coagulation(DIC),anti-neutrophil cytoplasmic antibodies(ANCA) did not show significant abnormalities. MRI of the head revealed multiple abnormal signal shadows in the left temporal lobe, as well as in the bilateral frontal, parietal, and occipital lobes, including subcortical and white matter regions, where blood and cortical lamellar necrosis were detected. High signal areas in the white matter were scattered throughout the bilateral frontal and parietal lobes, as well as near the lateral ventricle. CNSV was considered (**Figure 1**). PET-CT showed decreased cortical metabolism in the right frontal, parietal, occipital, and left temporal lobes. No abnormalities or signs of tumors were found throughout the body (**Figure 2**). On November 29, 2023, the neurosurgeon performed a left occipital lobe lesion resection. The histopathological biopsy revealed multiple tumor-like cell aggregations in the artery (**Figure 3**), and immunohistochemistry showed positive results for CD19, CD20, CD34, Pax-5, Bcl-2, Bcl-6, Mum-1, Ki-67. These findings support a diagnosis of large B-cell lymphoma, classified as non-GCB according to Hans classification (**Figure 4**). The final diagnosis was intravascular large B-cell lymphoma of the central nervous system, and the patient was transferred to the tumor hospital for further treatment. During the follow-up, the patient was treated with rituximab. At present, the urine protein has turned negative, and the Cr has returned to normal.

## 3 Discussion

IVLBCL is a rare and highly aggressive non-Hodgkin’s lymphoma. The average age of onset is 70 years, with a slight male predominance (1). It was first described in 1959 by an Austrian dermatologist in a skin biopsy (2). IVLBCL is defined as a specific subtype of extranodal diffuse large B-cell lymphoma in the 2016 Classification of Hematological and Lymphoid Tumors (3). IVLBCL primarily presents in two clinical forms. The first is the classic variant, which affects the central nervous system and skin. The second is a hemophagocytic variant associated with hemophagocytic syndrome, often involving the liver and spleen, as well as causing cytopenia (4). The central nervous system is involved in 35% of IVLBCL patients, with the most common manifestations including sensory and motor disorders, meningitis, sensory abnormalities, and aphasia (5). Additionally, it can be complicated by other organs damage, such as nephrotic syndrome due to kidneys involvement, and respiratory failure due to lung involvement (6). IVLBCL may be masked or imitated, the clinical and imaging presentation of IVLBCL can resemble multiple different diseases, including CNSV and multiple sclerosis(MS) etc., which make a definitive diagnosis challenging (7, 8). Currently, the gold standard for diagnosing the disease remains pathological biopsy, including brain and skin tissue biopsy. Immunohistochemical staining of CD20 and CD34 is particularly useful for diagnosis (6, 9).

The pathogenesis of the disease remains unclear, despite significant breakthroughs in histopathology at the molecular level. Tumor cells accumulate in the vascular lumen primarily because large B cells home to and are captured in the vascular environment. Abnormal aggregation often results from the expression of adhesion molecules, such as integrins and selectins, which enhance the adhesion of B cells to endothelial cells. An imbalance in the migration and transport pathways of large B cells results in their capture within vascular channels (10, 11). This process may be influenced by the absence of CXCL12 and CXCL9 expression in endothelial cells (12). Shimada K found that the rearrangement of PD-L1/PD-L2 resulted in immune evasion of PD-L1/PD-L2 overexpression (13), the results of the Gonzalez-Farre B study confirmed that b cell receptor/nuclear factor-κB signaling is associated with mutations in the immune escape pathway of IVLBCL (14).

Serological abnormalities associated with IVLBCL may include elevated levels of CRP, thrombocytopenia, anemia, hypoproteinemia, as well as increased Cr and bilirubin levels (15). An examination of CSF may show increased levels of protein and nucleated cells (16). Cerebrospinal fluid is abundant in circulating tumour DNA(ctDNA), which can detect tumor markers and monitor the therapeutic effects of chemotherapy in patients (17, 18), Bobillo S proposed that in a study of 19 patients with B-cell lymphoma involving the central nervous system, the detection of ctDNA in cerebrospinal fluid is superior to that in plasma and can predict and identify tumors earlier than MRI (19). MRI offers greater diagnostic value in imaging. A multi-case follow-up identified four main manifestations: (1) hyperintense lesions in the pons on T2-weighted imaging (T2WI), (2) nonspecific white matter lesions, (3) infarct-like lesions, and (4) meningeal thickening or enhancement (20). PET-CT serves as an effective imaging tool for disease diagnosis and provides anatomical guidance for stereotactic tissue biopsy. An early study on IVLBCL found that, in newly diagnosed lymphoma patients, FDG uptake in low-grade lymphoma was lower than in high-grade lymphoma. However, the diagnostic criteria for intravascular large B-cell lymphoma may result in missed diagnoses (21).

In this case, the patient exhibited stroke-like symptoms, including hemiplegia, paresthesia, and vertigo, along with headache and proteinuria. Despite treatment for cerebral infarction, the patient experienced recurrent attacks. Upon admission, signs of hemiplegia, vertigo, and ataxia were present. Although the head MRI findings can account for the symptoms mentioned, the patient’s stroke risk factors do not fit the common Trial of ORG 10172 in Acute Stroke Treatment (TOAST) classification of cerebral infarction. Consequently, we suspect that the patient may have a rare condition known as CVSV. CNSV is an inflammation of the vascular walls in the central nervous system, which causes vascular injury and can result in stenosis, occlusion, or thrombosis, ultimately leading to ischemic damage (22). To differentiate from CNSV, Yunce M reported a case of bilateral multiple strokes, diagnosed as central nervous system intravascular lymphoma via biopsy (23). Both diseases exhibit similarities in MRI, including microbleeds and vascular enhancement. Diagnosing and distinguishing the two diseases using cranial MRI is challenging. Yaura K and Kimura M reported a case of multiple intracranial microbleeds, suggesting that patients with such findings should also be suspected of IVLBCL (24, 25). In a small sample study, IVLBCL microbleeds and intravascular thrombosis were identified as distinct potential major imaging manifestations, with some patients displaying characteristic imaging on Susceptibility-weighted imaging(SWI) that hold diagnostic significance (26). IVLBCL still has a very small part of extravascular infiltration in the central nervous system, but the specific mechanism is not clear. Poropatich K reported that five patients with IVLBCL had extravascular invasion and proposed that IVLBCL has a potential tendency to form extravascular lesions in the central nervous system. It is not only a clinically heterogeneous entity but also a histologically heterogeneous one (27). In this case, combined with cranial MRI and PET-CT, neurosurgeons considered that there may be a tumor mass in the occipital lobe, leading to the selection of this site for resection.

In head MRI, IVLBCL can exhibit vascular enhancement. Some researchers suggest that the infiltration of lymphocytes and macrophages into the vascular wall during central nervous system vasculitis may explain the enhancement of the vascular wall. However, in IVLBCL, the vascular wall is typically unaffected. Therefore, it is suggested that infiltration primarily occurs around blood vessels, and vascular damage may take place in small-diameter vessels (28). Schaafsma JD reported a case in which vessel wall magnetic resonance imaging (VW-MRI) showed concentric circle enhancement of blood vessels, mimicking CNSV. It is proposed that the enhancement of the vascular wall results from the physical force of proliferating cells dilating the vascular lumen, which increases endothelial permeability and leads to secondary arterial wall edema and intravascular gadolinium leakage into the vascular wall. Another possibility is tumor-associated vascular wall damage, despite the high variability in the degree of vascular enhancement in IVLBCL proposed later after biopsy (29, 30). Similarly, small blood vessels are rarely enhanced in CNSV. For CNSV with suspected small blood vessels, tissue biopsy is preferred, and VW-MRI is not reliable for assessing the degree of enhancement of small blood vessels at the distal end (31). Aqeel F reported a patient with recurrent ischemic stroke who was initially diagnosed with central nervous vasculitis by brain biopsy. Subsequently, a large amount of proteinuria occurred during one year of treatment. The ratio of urinary protein to creatinine was 500 mg/g, which manifested as nephrotic syndrome. A renal biopsy revealed that the condition was IVLBCL, which was CD20 negative but positive for CD45, Mum-1, CD79a, and Ki-67. CNSV may be a manifestation of IVLBCL involving the central nervous system (32). IVLBCL involving the kidney and confirmed by renal biopsy is rare, this has been noted in 5% of cases (33). A review of IVLBCL with renal involvement exhibited infiltration of the malignant cells within the glomerular capillaries, with or without peritubular and interstitial involvement. The most common renal manifestation is proteinuria (90.0%), followed by renal failure (60.5%) and nephrotic syndrome (21.0%) (34). A multi-institutional, retrospective review of kidney biopsies and autopsies with a diagnosis of kidney IVLBCL reported the most common pathological manifestations were the glomerular infiltration by neoplastic lymphoid cells, the infiltration of arteries or veins and peritubular capillary were also exists. The spectrum of glomerular involvement may range from minimal mesangial hypercellularity, segmental infiltration peritubular capillaries to global glomerular infiltration and segmental podocyte foot process effacement. The invasion of lymphoma cells leads to different degrees of obstruction of glomerular capillary plexus, resulting in different clinical symptoms or symptom clusters, the most common renal manifestations was acute kidney injury (AKI), proteinuria, hematuria. Nephrotic-range proteinuria, nephrotic syndrome, secondary minimal change disease, secondary membranous nephropathy, and immune complex membrane proliferative glomerulonephritis may be occur in different patients (35). The mechanism of proteinuria in tumors involving the kidney may be tumor cells gathered in glomerular capillaries and peritubular capillaries, which can lead to vascular obstruction and ischemia, ischemia can prompt tubular cell injury, necrosis and immune inflammation. Glomerular structural changes including endothelial cell damage and podocyte disappearance, these changes would contribute to the proteinuria observed. Tubular injuries can also lead to the leakage of low-molecular-weight proteins, resulting in proteinuria. Moreover, the proteinuria seemed to increase at a relatively gradual rate among the observed patients (36). Our patient was diagnosed with IVLBCL by brain biopsy. We did not perform a renal biopsy on the patient and speculated that renal was involved caused by intravascular lymphoma involving the kidney based on the current therapeutic effect.

In this case, PET-CT indicated that there was a reduced metabolic activity in the lesion. There are three possible explanations for this observation. First, the number or density of living lymphoma cells within the vascular lumen may be low. Second, lymphoma cells may proliferate outside the blood vessels. Extravascular infiltration may go undetected if only limited tissues are examined. The third reason may be that the spatial resolution of PET is relatively low (37, 38); Zhao D performed PET-CT examinations on 42 patients with intravascular lymphoma; 31 (73.8%) showed FDG avidity, while 11 showed non-FDG avidity. Comparing the two groups revealed that the Ki-67 index was higher in the non-FDG avid group, suggesting that lymphoma cells proliferated at a faster rate. The higher index may be due to changes in the glycolysis rate of malignant cells (39).

The diagnosis of IVLBCL relies on pathological biopsy, which can be performed at various sites. In the current study, Random Skin Biopsy (RSB) significantly aids in diagnosing IVLBCL. Matsue K found that random skin biopsy and bone marrow biopsy were equivalent to continuous skin biopsy and bone marrow biopsy in 12 patients with IVLBCL. Additionally, random skin biopsy demonstrated superior diagnostic effectiveness (40). A retrospective analysis revealed that the sensitivity and specificity of RSB for diagnosing IVLBCL were 77.8% and 98.7%, respectively (41). It has been suggested that skin biopsy should be performed before brain biopsy in patients with suspected brain IVLBCL (42). While skin biopsy is generally less invasive and harmful compared to biopsies from other sites, its effectiveness depends on the location, number, and depth of the samples collected (43). In the Chinese guidelines, for patients with unexplained fever, after excluding other diseases, multiple multi-point biopsies of suspicious sites should be performed as soon as possible. It is emphasized that the muscular layer should be reached; sampling sites are recommended to be on both sides of the thigh and abdomen, which is of great significance for the early diagnosis of clinically suspected patients. The evidence suggests that when there is organ dysfunction, the affected site should be biopsied (44).

## 4 Conclusion

We suggest that IVLBCL should be considered in patients who present with recurrent stroke-like symptoms and fluctuating neurological deficits. Brain MRI findings of multiple cerebral infarctions, microbleeds, and meningeal enhancement, along with involvement of other organs, may support this suspicion. Additionally, CNSV may indicate a transitional manifestation of the disease. Diagnostic tools such as SIL2R, ctDNA in cerebrospinal fluid, multi-sequence MRI, and PET-CT may be useful for diagnosing the disease. Pathological biopsy is the gold standard for diagnosis. While random skin biopsies demonstrate high specificity and sensitivity, the sampling site should be determined based on the specific clinical situation when there is clear evidence of organ involvement.

## 5 Limitations

In this case, the patient’s head MRI examinations have been conducted multiple times, but the images remain unclear, preventing a clear depiction of the disease’s progression. Additionally, because the lesion is near the distal cortex and due to technological limitations, we have not been able to complete VW-MRI. We completed a biopsy of the affected brain tissue, but did not perform a biopsy of the kidney. No abnormalities were found in the patient’s skin, and a skin biopsy was not conducted. We speculate that the kidney is also affected, given the complete remission of proteinuria following treatment for IVLBCL.

## Data Availability

The datasets presented in this article are not readily available because of ethical and privacy restrictions. Requests to access the datasets should be directed to the corresponding author/s.
